# Supplementary figures and images for: Anterior chamber depth in mice is controlled by several quantitative trait loci
Source: PLoS One. 2023 Aug 25;18(8):e0286897. doi: 10.1371/journal.pone.0286897 (PMC10456175; doi:10.1371/journal.pone.0286897)

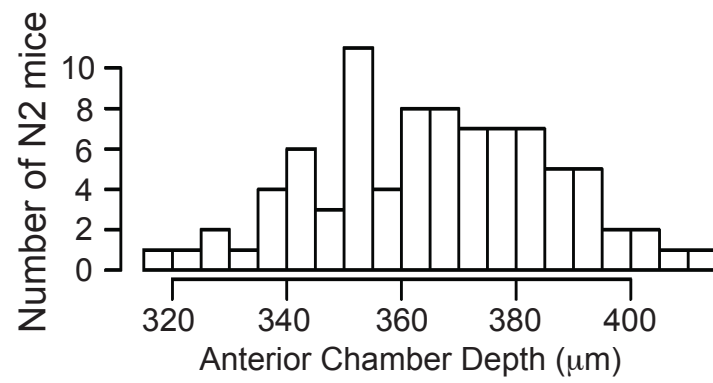

Supplement: S1 Fig — (PDF) [file pone.0286897.s001.pdf]

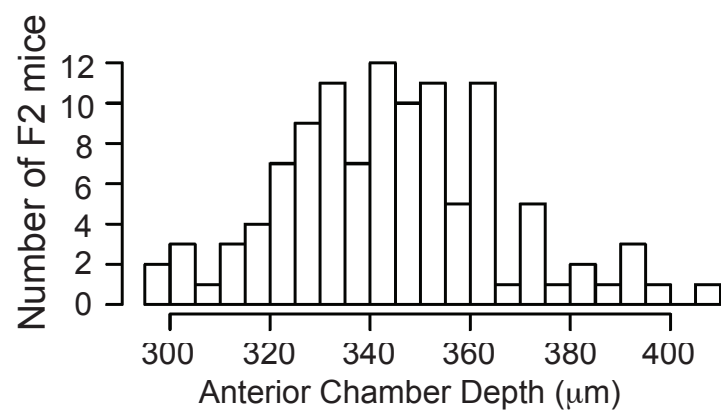

Supplement: S2 Fig — (PDF) [file pone.0286897.s002.pdf]
